# Fibroblast Activation Protein Alpha (FAPα) in Fibrosis: Beyond a Perspective Marker for Activated Stromal Cells?

**DOI:** 10.3390/biom13121718

**Published:** 2023-11-29

**Authors:** Nataliya Basalova, Natalya Alexandrushkina, Olga Grigorieva, Maria Kulebyakina, Anastasia Efimenko

**Affiliations:** 1 Institute for Regenerative Medicine, Medical Research and Educational Centre, Lomonosov Moscow State University, 119192 Moscow, Russiagrigorievaoa@my.msu.ru (O.G.); efimenkoay@my.msu.ru (A.E.); 2 Faculty of Medicine, Lomonosov Moscow State University, 119192 Moscow, Russia; coolebyakina@gmail.com

**Keywords:** fibroblast activation protein α (FAPα), fibrosis, activated fibroblast, myofibroblast

## Abstract

The development of tissue fibrosis is a complex process involving the interaction of multiple cell types, which makes the search for antifibrotic agents rather challenging. So far, myofibroblasts have been considered the key cell type that mediated the development of fibrosis and thus was the main target for therapy. However, current strategies aimed at inhibiting myofibroblast function or eliminating them fail to demonstrate sufficient effectiveness in clinical practice. Therefore, today, there is an unmet need to search for more reliable cellular targets to contribute to fibrosis resolution or the inhibition of its progression. Activated stromal cells, capable of active proliferation and invasive growth into healthy tissue, appear to be such a target population due to their more accessible localization in the tissue and their high susceptibility to various regulatory signals. This subpopulation is marked by fibroblast activation protein alpha (FAPα). For a long time, FAPα was considered exclusively a marker of cancer-associated fibroblasts. However, accumulating data are emerging on the diverse functions of FAPα, which suggests that this protein is not only a marker but also plays an important role in fibrosis development and progression. This review aims to summarize the current data on the expression, regulation, and function of FAPα regarding fibrosis development and identify promising advances in the area.

## 1. Introduction

The normal structure and function of various organ tissues can be disturbed when exposed to a wide range of damaging stimuli, such as viruses, chemical exposure, excessive immune reactions, or mechanical injury. Restoration of the lost tissue structure occurs in the process of reparative regeneration. During tissue repair, damaged or dead cells are removed, and the damage site is closed with temporary granulation tissue, characterized by a large number of immune cells, immature newly formed vessels, as well as cells actively synthesizing components of the extracellular matrix (ECM)—myofibroblasts [1,2]. Subsequently, granulation tissue is gradually replaced by cells specific to the tissue during the remodeling process. It should be noted that in adult mammals, often only the partial restoration of functional tissue is possible, with the preservation of connective tissue rich in ECM components and myofibroblasts.

However, chronic damage can lead to the formation of a tissue microenvironment that will constantly support repair processes [3,4,5]. The result of this exposure is the development of a condition called progressive fibrosis or, in medical terms, fibroproliferative disease. This condition is characterized by a gradual progressive replacement of the functional tissue of an organ with connective scar tissue, which can ultimately lead to organ dysfunction and even to the death of the organism [6,7].

As mentioned above, one of the pivotal cell types that mediate both fibrosis development and progression is myofibroblasts [5,8]. Indeed, myofibroblasts, the main marker of which is alpha-smooth muscle actin (αSMA), are responsible for the high rate of ECM remodeling due to their secretory activity (synthesis of ECM components like fibronectin and fibronectin containing extra domain A (EDA-fibronectin), collagen I and III, IV, VI type, hyaluronic acid, and periostin) [9,10,11] and capacity for ECM contraction, which leads to an increase in its stiffness [12,13]. Moreover, part of the molecules secreted by myofibroblasts promote the activation of surrounding cells and their further transdifferentiation into myofibroblasts. Permanent transdifferentiation creates a positive feedback loop and the further progression of fibrosis [3,13,14,15,16].

For a long time, myofibroblasts were considered a key cellular target in studying fibrosis mechanisms and developing antifibrotic therapeutic agents. At the moment, there are various approaches aimed at eliminating myofibroblasts from tissue: the inhibition of inflammation or individual proinflammatory factors [17,18,19,20], the induction of myofibroblasts apoptosis [21,22,23], and the inhibition or even reversal of fibrosis by inducing the transdifferentiation of functional myofibroblasts into tissue-specific cell types, such as lung lipofibroblasts [21,24,25,26,27,28]. Despite the effectiveness of these approaches in vitro or even in animal models in vivo, they have demonstrated very limited success in clinical practice [29,30,31,32,33,34,35]. It should be noted that for the treatment of some fibroproliferative diseases, such as idiopathic pulmonary fibrosis (IPF), few effective drugs are available, like pirfenidone and nintendanib [34,36,37]. However, the rather high cost (about USD 30,000 for an annual course throughout the life) as well as the significant side effects limit their availability to patients. In addition, these drugs are not able to completely inhibit progressive fibrosis and, even more so, are not able to ensure the restoration of the original structure and function of the tissue. Therefore, close attention in the search for drivers of fibrosis has been attracted by populations that may be more labile and responsive to therapeutic attempts compared to myofibroblasts.

## 2. The Overview of “Activated Stromal Cells” and FAPα as Their Marker

A promising area of study in recent years has been a cell population that appears to be the precursor of myofibroblasts and is called “activated stromal cells”. For the first time, the concept of the presence in tissue of cells preceding myofibroblasts was formed in the 1970s–1980s. Such cells have been termed “competent” or “activated” because of their ability to rapidly respond to changes in ECM composition or growth factors such as platelet-derived growth factor (PDGF) by increasing proliferative activity and secreting matrix proteins [38,39]. The idea that these cells could be responsible specifically for the progressive form of fibrosis was initially pointed out in these articles [40,41]. According to current concepts, a myofibroblast is predominantly formed from a fibroblast, or, in a broader sense, a tissue-specific stromal cell, through the stage of an activated cell (proto-myofibroblast) [42,43]. The appearance of activated cells is induced by damage-associated secretory factors such as platelet-derived growth factor (PDGF) as well as by mechanical stress. Thus, PDGF stimulates proliferation, type I collagen production, and also the motility of late proto-myofibroblasts [9,44,45]. The main properties of activated cells include high migratory activity and proliferative levels, as well as a high level of ECM secretion, including type I collagen and EDA-fibronectin [43]. Differentiated myofibroblasts can be distinguished from proto-myofibroblasts by the de novo expression of αSMA and the increased expression of EDA-fibronectin, as well as the increased assembly of αSMA-stress fibrils and supermature focal adhesions [42,46].

In vivo, in fibrosis of the lungs and some other organs like the heart, liver, pancreas, and breast, the main morphofunctional unit is a fibrotic focus (fibroblastic focus) [47,48,49,50]. Low-proliferating, actively expressing αSMA myofibroblasts, surrounded by ECM, constitute the myofibroblast core of the focus, capable of self-maintenance. The second important part of the fibroblastic focus is the active fibrotic front along the periphery of the myofibroblast core [51,52]. Because this zone is defined as a “highly cellular and mitotically active region” [52], this is probably the key place where fibroblasts become activated. Thus, an activated fibroblast may contribute to an increase in the area of the fibroblastic focus due to contact with the core ECM leading to constant proliferation and rapid transdifferentiation into myofibroblasts. Moreover, due to the high migration activity mediated by the expression of the hyaluronic acid receptor CD44, metalloproteinases (MMP) 2 and 9, such cells are able to invade normal tissues and thereby increase the area of the affected tissue and the number of fibroblastic foci, i.e., promoting the progression of fibrosis [53,54]. It should be emphasized that the development of a fibrotic focus apparently begins with the appearance of activated cells within the tissue, which are usually located close to the basement membrane of the damaged epithelium and can be activated directly by signals coming from damaged epithelial cells [53,55,56]. Therefore, activated stromal cells could serve as a pivotal driver of fibrotic niche formation and promote fibrosis progression.

However, at the moment, there is no clearly established and recommended marker that would allow for isolating activated cells. Studies in this field have led to the discovery of a surface antigen that is difficult to detect in healthy adult organs but significantly increased at sites of tissue remodeling, including the periphery of the myofibroblast core within fibrotic tissue [57,58,59]. This antigen is called fibroblast activation protein alpha—FAPα. In the 1990s, a group of scientists led by Chen demonstrated that the cell surface expression of FAPα had significant effects on cell motility, ECM degradation, and invasive behavior [58]. Thus, FAPα seems to be a promising marker for activated stromal cells in fibrosis. However, important questions remain open: is FAPα only a marker or does it play an important role in the functioning of activated stromal cells during fibrosis? Could FAPα-positive cells be the proper target for the therapy of fibroproliferative diseases? Further in this review, these issues will be elucidated in detail.

## 3. FAPα as a Protein—Structure, Activity, Localization

**Figure 1:**
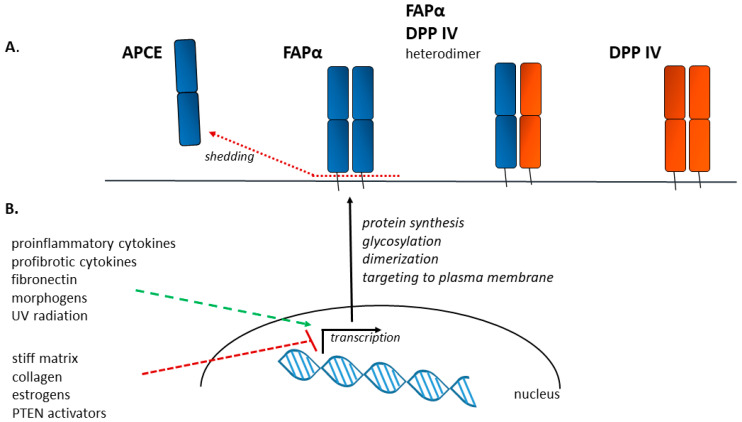
Factors influencing the expression of FAPα. (**A**). On a cell membrane, the main form of FAPα (blue) with enzymatic activity is a homodimer. The APCE (antiplasmin cleaving enzyme) protein, formed as a result of FAPα shedding, has similar enzymatic activity. The closest homolog of FAPα is DPP IV (CD26, orange) protein, which may form heterodimers with FAPα. (**B**). The amount of FAPα in cells is positively affected by some proinflammatory and profibrotic cytokines (e.g., TGFβ, TNFα, IL-1β), fibronectin, morphogens (retinoic acid and bFGF), or UV radiation. Negative regulators of FAPα expression include a stiff matrix (≈20 kPA), collagens, estrogens, and PTEN activators. After transcription and protein synthesis, glycosylation and dimerization steps are required for the proper insertion of FAPα into the membrane.

Structurally, FAPα is a transmembrane glycoprotein with a molecular weight of 95–105 kDa (of which *N*-glycosylation accounts for about 20 kDa). FAPα is localized predominantly in invadopodia, membrane regions associated with the degradation of the ECM during cell migration and invasion. The C-terminal region of the FAPα molecule, exposed on the external side of the plasma membrane, has dipeptidyl peptidase and protease activity; thus, the second name of FAPα is seprase (surface expressed protease) [60,61]. Enzymatic activity requires the glycosylation of the FAPα protein and depends on its dimerization [62]. The existence of a soluble form of FAPα, formed as a result of shedding, was also shown; this form is called APCE (antiplasmin cleaving enzyme) and exhibits the same substrate specificity as membrane-bound FAPα [61,63] (Figure 1A).

FAPα is capable of hydrolyzing a set of substrates, including proteins of the ECM and biologically active factors deposited in it, membrane-bound proteins, and soluble molecules of a peptide nature [64]. Among the substrates of FAPα endopeptidase activity type I and III collagens, alpha-2-antiplasmin (α2AP) and fibroblast growth factor 21 (FGF-21) have been well studied. The substrates of the dipeptidyl peptidase activity of FAPα are neuropeptide Y, peptide YY, substance P, and brain natriuretic peptide [65]. Available data on the specificity of FAPα enzymatic activity make it possible to test whether a protein of interest would be a potential substrate of a given peptidase based on its primary sequence [66].

Of the group of membrane-bound proteases to which FAPα belongs, dipeptidyl peptidase 4 (DPP4), or CD26, is most similar to FAPα [67]. Despite the similarity in structure and the fairly high (48%) homology of primary sequences, FAPα and CD26 differ significantly in properties and expression in tissues. Thus, CD26, unlike FAPα, is widely expressed in normal tissues. Generally, the expression patterns of FAPα and DPP4 in the organs overlap very little, with the exception of invading fibroblasts and some tumor cells. The factors regulating the expression of DPP4 and FAPα differ greatly [68]. In addition, it is worth noting the differences in the substrate specificity of FAPα and DPP4: according to recent studies, DPP4, unlike FAPα, does not have endopeptidase activity [69]. Thus, despite the high similarity of these proteins, allowing some predictions about their properties to be made (for example, several substances previously identified as substrates of DPP4 were also substrates of FAPα), there are many differences between DPP4 and FAPα, which makes such predictions very limited and requires experimental verification.

In the body, the transient expression of FAPα is observed in some tissues of the embryo of mesenchymal origin, but in the tissues of an adult organism, its expression is practically absent (none or very low). Exceptions include some types of cancer, wound healing, and fibrosis [53,70]. FAPα-deficient mice (FAPα^−/−^) are viable and show no obvious developmental defects [71]. Joachim Neidermeyer et al. replaced the FAPα gene with β-galactosidase, which was regulated by the FAPα gene promoter. The replacement of FAPα with β-galactosidase did not result in obvious changes in the phenotype, suggesting that FAPα is associated with tissue remodeling but is not strictly required in embryonic development. The activation of compensatory proteolytic enzymes may promote normal development in FAPα-deficient models [72]. Considering that wound healing is often accompanied by a repetition of a number of cellular events of embryonic development [73], it is reasonable to assume that the expression of FAPα in fibroblasts observed during wound healing has physiological significance.

Low basal levels of FAPα expression in healthy mice can be found in bone marrow, adipose tissue, skeletal muscle, and skin [74]. According to the authors’ observations, FAPα is also detected in stromal cells in the lungs of healthy mice in single cells [24]. In humans, FAPα RNA is also observed in the endometrium [75]. At the same time, FAPα is detected in the reactive stroma in stromal cells of almost all solid tumors, as well as in sites of tissue remodeling due to chronic inflammation, fibrosis, or wound healing. With the development of fibrosis, the proximity of FAPα cells to the epithelium is noted [53]. These data correlate with the modern concept that the development of fibrosis can begin directly with the activation of fibroblasts from the damaged epithelium, without affecting the immune cells at the earliest stages [55]. Moreover, FAPα expression often directly correlates with the severity of inflammatory reactions and edema [76], returning to normal levels as these processes resolve. However, it should be noted that FAPα expression in inflammatory cells, endothelial cells, or vascular smooth muscle cells is rare or observed at a very low level [76,77,78].

## 4. Factors Influencing FAPα Expression

Despite the abundance of information on the role of FAPα in the pathogenesis of a number of tumors, there is relatively little data on the factors regulating FAPα expression in fibroblasts, including fibrotic processes (Figure 1B). The work of Rettig et al. showed that exposure to retinoic acid led to an increase in the expression of FAPα in cultured fibroblasts [79]. Retinoic acid is an important morphogen; it is probable that FAPα is induced during embryogenesis under its influence. In addition to retinoic acid, FAPα increases in fibroblasts under the influence of transforming growth factor beta (TGF-β) and the 12-O-Tetradecanoylphorbol-13-acetate (TPA), and, apparently, this occurs through independent mechanisms: the effects of TGF-β and TPA on FAPα expression are cumulative. According to the results of inhibitory analysis, the effect of TGF-β on FAPα expression can be carried out through the triggering of the canonical signaling pathway (mediated by the phosphorylation of SMAD2/3) and is independent of ERK/MAPK [78]. In addition to TGF-β, the increased expression of FAPα in cultured fibroblasts is also caused by exposure to the morphogen basic fibroblast growth factor (bFGF), Interleukin 1 beta (IL-1β), tumor necrosis factor alpha (TNFα), and Interleukin 4 (IL-4) [79,80,81]. Despite the available data, in Avery’s work, none of the more-than-profibrotic factors, including Interleukin 3 (IL-3), interferon gamma (IFNγ), Interleukin 6 (IL-6), TNF-α, and TGF-β, except for ascorbic acid, did not increase the expression of FAPα in mouse lung fibroblasts. However, many studies assign a key role to TGF-β in determining the ratios of FAPα^+^ and αSMA^+^ cell populations [59,78,82,83]. It can be suggested that PDGF should be another factor activating FAPα expression, but precise evidence for this has not yet been published.

Changes in the culture substrate composition and Young’s modulus also affect FAPα expression. Thus, it has been shown that an increase in the stiffness of the culture plastic inversely correlates with the expression of FAPα. However, if collagen is used as a substrate, FAPα expression decreases regardless of the stiffness substrate. Conversely, the use of fibronectin or the matrix of decellularized cell sheets as a substrate increases both the expression and the amount of FAPα protein in cells [82].

Interestingly, the nonspecific inhibition of dipeptidyl peptidases, including FAPα, in fibroblast-like synoviocytes leads to a decrease in FAPα expression [84], which may indicate the existence of a positive feedback loop in which FAPα in fibroblasts helps maintain its own expression. This may be mediated through autocrine action, as it has recently become known that FAPα-positive fibroblasts express and secrete significantly more proinflammatory cytokines (see the chapter “Paracrine Activity” below). It has also been shown that FAPα expression is increased in fibroblasts exposed to ultraviolet radiation [85].

Negative regulators of FAPα expression include estrogens and activators of PTEN protein [86,87]. Importantly, the FAPα promoter region is known to contain binding sites for several transcription factors—AREB6, ITF-2, Meis-1, PPAR-gamma1, PPAR-gamma2, and Tal-1beta [88]. However, the effect of these proteins on FAPα expression has yet to be investigated. In addition to expression, the regulation of FAPα functioning can be mediated by changing its enzymatic activity. No specific endogenous activators or inhibitors for FAPα have been identified. However, since the nonglycosylated form of FAPα is known to lack enzymatic activity [62], the deglycosylation of FAPα can be considered as a way to regulate its activity.

## 5. Functions of FAPα- and FAPα-Positive Cells in Fibrosis

Available data allow for recognizing several mechanisms through which FAPα may influence the development of fibrosis. First of all, these are mechanisms associated with the enzymatic activity of FAPα: the processing of bioactive proteins (growth factors, chemokines, and hormones), which normally contributes to their degradation; the degradation of ECM components (primarily the further breakdown of collagen types 1, 3, and 4 degradation products); and the regulation of the activity of other enzymes (both membrane-bound and soluble) through limited proteolysis. The abovementioned functions can be realized by FAPα in the near-membrane region or at a distance from the cell, as a result of FAPα shedding. In addition, FAPα may interact with other membrane proteins and regulate their functions through mechanisms unrelated to the enzymatic activity of FAPα. Cells positive for FAPα could also contribute to the development and progression of fibrosis due to several mechanisms. Below, these possible mechanisms will be considered in more detail.

### 5.1. Paracrine Activity

The data obtained for cancer-associated fibroblasts (CAFs) suggest that FAPα-positive fibroblasts have a specific secretory profile. In particular, these cells secrete significantly more cytokines (CCL2 and IL-6) and cell adhesion molecules (CXCL8) [89]. FAPα-positive decidual fibroblasts are characterized by high levels of expression of a number of chemokines (such as growth-related oncogene alpha, CCL2, and monocyte chemoattractant protein-2 (MCP-2)), proinflammatory cytokines (IL-1α and TNF-α), and positive regulators of angiogenesis (primarily vascular endothelial growth factor (VEGF), angiogenin (Ang), bFGF, and hepatocyte growth factor (HGF)) [90,91]. Thus, through paracrine activity, FAPα-positive fibroblasts can regulate not only invasion and migration but also the proliferation of other cells, as well as the inflammatory response. However, a detailed characterization of the secretome of FAPα-positive fibroblasts in fibrosis is currently absent.

FAPα expression in tissue correlates with increased angiogenesis and increased capillary density, according to numerous observations in tumor models, but data on fibrosis are currently lacking [92,93]. The shedding of FAPα and the appearance of its soluble form does not lead to a change in the enzymatic activity of FAPα but allows FAPα to diffuse in the intercellular space and act at a much greater distance from the primary cell [61,63]. The possible role of FAPα in angiogenesis should also be noted, which is not associated with the paracrine activity of this molecule. Membrane heterodimeric complexes of FAPα and DPPIV on endothelial cells have been shown to facilitate the degradation of the collagen matrix and thus promote endothelial cell migration [94]. 

Thus, by analogy with FAPα-positive cancer cells, it may be assumed that FAPα-positive cells in fibrosis have a specific secretory phenotype, including the increased production of proinflammatory cytokines and angiogenesis regulators. However, studies of the secretion profile of fibrosis-associated FAPα-positive cells are strictly required to accurately characterize their secretome.

### 5.2. ECM Remodeling

Fibroblasts expressing FAPα induce architectural and compositional changes in ECM primarily by modulating the levels of fibronectin and collagen production, as well as by changing their structural organization [95]. As shown by zymography, FAPα can cleave gelatin and human type I and III collagen, partially cleaved by other metalloproteinases, but was unable to cleave human fibronectin, laminin, or type IV collagen [96,97]. FAPα is not capable of breaking down native collagen: its substrates are only denatured and partially degraded collagen (so-called gelatinase activity). Thus, FAPα is not the initiating enzyme of the ECM proteolysis process but rather an accelerator of its degradation, working with collagen hydrolysis products formed by other proteases. Proteolysis catalyzed by FAPα leads to the cleavage of a 12 amino acid residue-long peptide from the N-terminal region of α2AP, and the resulting α2AP derivative is able to cross-link with fibrin an order of magnitude faster than the original α2AP molecule [63,98]. In addition, Avery’s work showed that the gelatinase activity of fibroblasts with a high level of FAPα expression was eight times greater than that of cells with a myofibroblast phenotype [82].

Interestingly, in FAPα knockout mice, signs of fibrosis are exacerbated, and the restoration of FAPα expression significantly reduces the amount of collagen in lung tissue. Based on this, it can be assumed that FAPα may play a protective role in the lungs by promoting collagen destruction and matrix degradation [98,99]. It was also shown that mouse lung fibroblasts with a high level of FAPα expression demonstrated a high expression of ECM proteolysis-associated genes—MMP1A, MMP2, MMP3, MMP8, MMP9, MMP12, MMP13, and TPA, with the exception of MMP14 and plasminogen activator (PLAU) [82,100,101]. However, the gene expression of most tissue inhibitors of metalloproteinases (TIMPs) was comparable or reduced in FAPα^+^ fibroblasts compared to αSMA^+^ myofibroblasts, with the exception of TIMP4 [82]. The inhibition of FAPα by antibodies results in the decreased secretion of TIMP-1, but not MMP-3 and -12, from ex vivo cultured human intestinal stenosis specimens [102]. At the same time, when an FAPα inhibitor is administered to mice with liver fibrosis, MMP-2, -9, -13, and TIMPs are decreased.

On the other hand, it has been well shown that during the fibrosis of various organs in vivo, FAPα-positive cells are surrounded by type I collagen and fibronectin fibers [100,102,103] and also express prolyl-4-hydroxylase β [78]. It is possible that such a feature is more characteristic of the late stage of fibrosis development [104]. It should also be noted that FAPα^+^ cells have a probable affinity for hyaluronic acid, since some studies have shown its colocalization with CD44 [100,105,106], which suggests that FAPα-positive cells are capable not only of active ECM degradation but also of the synthesis of ECM proteins. Thus, FAPα^+^ fibroblasts showed a higher gene expression of many components of ECM, including type I and III collagens, decorin, EDA-fibronectin, thrombospondin-2, and osteopontin [82].

However, in vivo, using FAPα knockout animals in an atherosclerotic plaque model, where FAPα deletion accelerates atherosclerosis, or an infarction model, no increase in either total or fibrillar collagen or fibronectin was found [77,107]. It should be noted that significant changes in the composition of the matrix were still noted in female mice—they had an increased area of type 1 collagen—as well as the intensity of its luminescence, but there was a decreased size of the fibronectin area [77]. In the model of CCl4-induced liver fibrosis, the amount and expression of type 1 and 3 collagens, as well as osteopontin, decreases with the introduction of the FAPα enzymatic activity inhibitor during the progression of fibrosis, but not during the remodeling stage [104].

Taken together, the accumulated data indicate the controversial role of FAPα in fibrosis-associated ECM remodeling. On the one hand, FAPα-positive cells have the ability to actively secrete ECM components, including collagen I and EDA-fibronectin, which are crucial for the progression of fibrosis. On the other hand, the high level of proteolytic protein expression, along with the enzymatic properties of FAPα, allows for the high proteolytic activity of FAPα-positive cells.

### 5.3. Migration and Invasion

Numerous studies confirm that the maintenance of the invasive fibroblast phenotype in fibrosis is strictly necessary for the development of fibrosis. Although the mechanisms by which invasive cells contribute to the development of fibrosis are not fully understood, in general, the inhibition of factors associated with the invasive phenotype of stromal cells leads to a decrease in the development of fibrosis [54,108,109,110]. Possible mechanisms may include an increase in the ability of cells to migrate to the site of injury [111,112], the destruction of the basement membrane and damage to epithelial cells [113,114], as well as the creation of new fibrotic foci in uninjured tissue [52,115].

The collagen-rich ECM plays a central role in regulating cell and tissue biology in various organs in health and disease. The above-described ability of FAPα to degrade collagen and remodel the ECM has a significant impact on the motility and invasive behavior of both stromal cells expressing FAPα and other cell types (endothelial, tumor cells, and others) [116]. Over the past two decades, the most active area of study of FAPα has been oncology, but the role of FAPα is also being actively studied in the pathogenesis of fibrotic diseases, including arthritis, IPF, atherosclerosis, and fibrotic conditions of the liver and colon. Thus, FAPα-dependent changes in ECM promote vascular and tumor cell invasion along the specific parallel orientation of collagen fibers, as evidenced by the increased targeting and velocity of cancer cells to the ECM from FAPα^+^ cells. This phenotype can be reversed by inhibiting the enzymatic activity of FAPα during matrix formation, which leads to the disorganization of the ECM and prevents tumor invasion.

As in the development of tumor pathologies, the prolonged expression of FAPα promotes the invasive growth of fibroblasts in fibroproliferative conditions such as keloids [117,118], and FAPα expression is increased eightfold in the deepest part of the keloid compared with that in healthy skin. FAPα-positive cells are also found in the infarcted area and tissue remodeling adjacent to the infarcted area [78,103], as well as in atherosclerotic plaques [77]. FAPα expression correlates with the expression of molecules responsible for the invasive state of the cell. Thus, it was found that the number of FAPα-positive cells correlates with the number of cells positive for SNAIL, CD44, HIC-5, and RAGE in the case of proliferative vitreoretinopathy [106].

In the article by Dienus, it was shown that fibroblasts isolated from an actively growing margin of keloid scars had a significantly high level of FAPα protein and invaded three times better than fibroblasts from normal areas of the skin. The inhibition of FAPα by H2N-Gly-Pro diphenylphosphonate (FAPα/DPPIV inhibitor) in keloid fibroblasts reduces their invasive activity to near normal levels without affecting the invasiveness of normal fibroblasts [118]. Similar data indicating an increase in the invasive activity of FAPα-positive cells were obtained with the overexpression of FAPα, including a mutant non-enzymatic form, in the immortalization of the primary human hepatic stellate cell line LX-2 [100]. On the other hand, the same article showed that HEK293T cells overexpressing FAPα and DPPIV have reduced invasive potential on various substrates, such as type I collagen, Matrigel, and fibronectin, compared to control cells (although it should be noted that in normal HEK293T cells, neither FAPα nor DPPIV are not expressed at all) [100]. A possible reason for the decrease in invasive properties is the decrease in adhesion to the type I collagen, Matrigel, or Matrigel substrates in cells overexpressing FAPα (but not DPPIV) or its enzyme-inactive mutants [102]. Interestingly, FAPα-positive fibroblasts from areas of human intestinal stenosis, on the contrary, increase their migratory activity when adding blocking antibodies [102].

### 5.4. Myofibroblast Differentiation

Another important mechanism for the contribution of FAPα in the development of fibrosis may be the direct regulation of cell differentiation into myofibroblasts.

It has been shown in the lung murine fibrosis model that the introduction of CAR-T-anti FAPα^+^ cells 6 weeks after the administration of bleomycin leads to a tendency toward a decrease in αSMA^+^ myofibroblasts [99]. On the other hand, in the same work, when modeling fibrosis in FAPα-knockout mice, the amount of αSMA was significantly greater than that in control mice [99]. In Crohn’s disease, which is a chronic intestinal inflammation that ultimately leads to fibrosis, an increased expression of FAPα has also been found [102,119]. However, the overexpression of FAPα was observed only in strictures (scarred areas) compared to non-stricture areas of the colon in biopsies taken from patients with Crohn’s disease. FAPα was not overexpressed in colon biopsies taken from healthy people or people with ulcerative colitis, another inflammatory bowel disease. In addition, upon exposure to TNFα and TGF-β, FAPα expression was increased in myofibroblasts derived from stricture lesions only, but not in myofibroblasts from nonstricture lesions [119]. These results imply that FAPα does not appear in all fibroblasts when exposed to inducing factors, which has also been shown for cirrhosis, in which FAPα is not expressed by all αSMA^+^ myofibroblasts, suggesting that FAPα marks a differentially activated state of fibroblasts [120]

Indeed, in most cases, FAPα and αSMA^+^ identify distinct, non-overlapping subsets of fibroblasts [121,122]. However, there are certainly overlapping FAPα^+^αSMA^+^ subpopulations—in the epiretinal membranes of patients suffering from proliferative vitreoretinopathy [106], in samples of intestinal stenosis [102], in the synovial membranes of rheumatoid arthritis [105], in a human liver with cirrhosis [100], and in skin samples from patients with scleroderma [123]. The expression and amount of FAPα are high in patients with both interstitial lung disease (ILD) and silicosis. In a bioinformatics analysis of a single-cell transcriptome of cells obtained from patients with ILD, it was shown that FAPα expression was observed only in clusters of myofibroblasts or fibroblasts with a high level of hyaluronan synthase 1 expression [59].

Since in some conditions, adipogenesis is considered to be a competitive process for the differentiation of fibroblasts into myofibroblasts, FAPα and its ability to regulate adipogenesis can also be considered one of the pathways regulating differentiation. It has been shown that FAPα is necessary in vivo for the proteolytic processing of the C-terminal region of FGF21, leading to the inactivation of the latter [124], which leads to metabolic dysfunction and obesity [125,126]. In addition, FAPα may be involved in the regulation of adipogenesis by interacting with Thy-1 (CD90), which binds to FAPα in lipid rafts and leads to the suppression of adipogenesis [127].

However, at the moment, there is a lack of understanding of how FAPα affects the differentiation of stromal cells into myofibroblasts. The exact role of specific subpopulations of FAPα-positive cells also remains unclear. Moreover, the conditions governing the phenotypic heterogeneity and functional role of these phenotypically distinct subsets of stromal cells are still unknown.

### 5.5. Immune Response

Studies of the expression profile of FAPα^+^ fibroblasts showed an increase in the expression level of genes of pro-inflammatory factors, such as TNF-α, IL-1β, IL-6, IL-18, and chemokines of the CCL and CXCL groups [76]. Synovial fibroblasts with a high expression of FAPα have been identified as key effector cells in the inflammatory disease of rheumatoid arthritis [128]. The synovial expression of FAPα was either low or undetectable at rest, increased significantly during the course of arthritis, and correlated with the severity of ankle swelling [129,130]. The deletion of FAPα^+^ cells resulted in a decrease in leukocyte infiltration, was negatively correlated with the severity of joint inflammation, and was associated with a decrease in the number of fibroblasts without a significant change in the number of pericytes.

One of the main types of immunomodulatory tissue cells is tissue macrophages. Modeling liver fibrosis and the subsequent suppression of FAPα in mice using a specific inhibitor (FAPi) showed a decrease in the level of collagen, αSMA^+^ myofibroblasts, ALT and AST levels, and key transcripts associated with fibrogenesis under the influence of the inhibitor. Histological analysis showed that during the development of fibrosis, FAPα^+^ cells were found at the border of fibrous septa in the liver in both mice and humans, next to macrophages. Moreover, FAPi administration led to a decrease in the number of macrophages, but not CD3^+^ lymphocytes in the liver [104]. The FAPα inhibitor also reduced the level of hepatic transcripts of the pro-inflammatory genes CCL2 and NOS2, which the authors associated with a decrease in macrophage levels. However, given the pro-inflammatory expression profile of FAPα^+^ fibroblasts themselves, it remains unclear whether and to what extent the effect of FAPi changes the expression profile of the FAPα^+^ cells. The direct effect of recombinant FAPα on bone marrow-derived M2 macrophages showed a change in their expression profile with increased levels of transcripts of both pro-inflammatory and anti-inflammatory factors. The overall profile indicated a switch from the M2 phenotype of macrophages to the pro-inflammatory M1 phenotype.

A study of the influence of factors secreted by HSP liver stellate cells showed that an HSP-conditioned medium after FAPi suppressed the transcription of pro-inflammatory factor genes in macrophages in vitro.

An analysis of FAPα levels in the lungs of mice after the intratracheal administration of bleomycin (BLM) showed an increase in its expression level, but the administration of PT100, an orally active dipeptidylpeptidase activity inhibitor, led to a decrease in FAPα expression in the lungs of BLM-treated mice. An immunohistochemical analysis of lung tissue on the 14th day after the last injection of saline or bleomycin showed that IBA-1 (a marker of macrophage activation) and CD3 (a marker of T lymphocytes) were significantly increased in BLM-treated mice compared to in mice injected with saline. Moreover, IBA-1 levels were significantly increased in PT100-treated animals, suggesting an increased number of activated macrophages in this group [131]. The authors note that the role of IBA-1 in inflammation includes the migration, proliferation, and activation of macrophages, as well as signal transduction [132]; however, further elucidation of the mechanism of the cross-interaction of FAPα^+^ fibroblasts and macrophages in the lungs is required. Unfortunately, the limited range of macrophage markers analyzed in the work does not make it possible to accurately determine the subtype of the analyzed cells.

The presented data allow us to conclude the importance of the role of the interaction of FAPα and FAPα^+^ cells with macrophages in the development of fibrosis and also suggest that FAPα inhibition may become a novel therapeutic approach to the prevention and treatment of fibrosis.

## 6. Cancer-Associated Fibroblasts (CAFs) and FAPα-Positive Fibrosis-Associated Cells (FAFs)—Obvious Similarity to Transfer the Conceptions from Cancer to Fibrosis

For a long time, FAPα has been mentioned only as a marker of cancer-associated fibroblasts (CAFs), which are an essential component of tumor stroma. CAFs surrounding the tumor are key cells influencing tumor growth and metastasis [66,133]. Moreover, FAPα is considered to be one of the major proteins mediating the functional activity of CAFs. However, the formation of CAFs is just a particular example of the fibroblast activation process occurring also during tissue damage and subsequent wound healing. This process includes the appearance of activated fibroblasts actively migrating to the damaged area, which are able to differentiate into myofibroblasts and secrete ECM proteins, forming the stroma of the newly formed tissue. During the last decades, CAFs have been extensively investigated (reviewed in detail in [118]), and this study does not aim to cover and thoroughly analyze these studies in the present review. However, it is required to highlight the similar features between CAFs and FAPα-positive stromal cells activated in fibrosis to speculate if these cells resemble each other in terms of both functional properties and mechanisms of contribution to the pathological processes.

**Figure 2:**
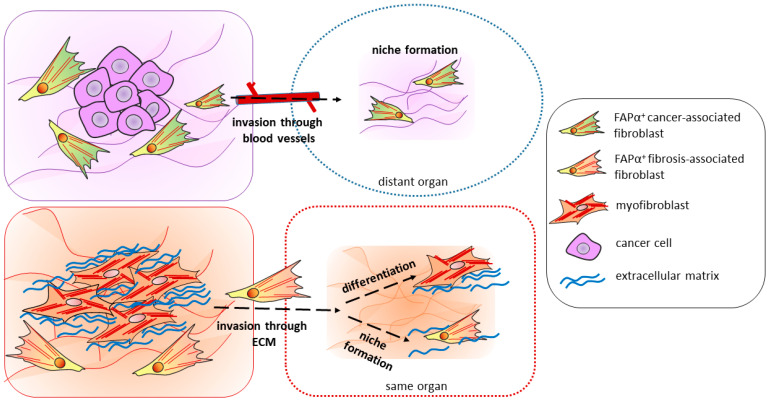
The mechanisms of fibrotic foci formation may be similar to those of tumor metastasis. Top: FAPα^+^ cancer-associated fibroblasts (CAFs) surround tumor cells, creating a favorable microenvironment for tumor growth. These cells are also capable of invasion through blood vessels into other organs. There, CAFs create a niche for tumor cell invasion, which leads to the formation of metastasis. Bottom: Similar to CAFs, FAPα^+^ fibrosis-associated cells (FAFs) surround the core of the fibrotic focus. Presumably, these cells are capable of direct invasion into healthy nearby tissues. In a new place, FAFs may give rise to a new focus both through the formation of a niche or differentiation into myofibroblasts—the main cells of the focus.

With the growth of connective tissue (desmoplasia), the remodeling of the local ECM microenvironment around the tumor site causes the mechanical compaction of tissues and their increased tension, which can be a signal for both the survival of CAFs and the activation and recruitment of resident fibroblasts. This process resembles the activation of new FAPα-positive stromal cells, particularly around the core of the fibrotic focus. The paracrine activation of fibroblasts, both in the case of tumor formation and during fibrogenesis, includes the action of the factors PDGF, TGF-β, IL-6, and connective tissue growth factor (CTGF) [134,135], and in both cases, tissue epithelial cells are considered as a source of activated fibroblasts due to the epithelial–mesenchymal transition (EMT). In addition, like CAFs during metastasis, FAPα-positive activated stromal cells are probably capable of active invasion into healthy tissue and the creation of new fibrotic foci there (Figure 2). Other important functions of both cell types for the progression of fibrosis include maintaining the invasiveness of surrounding cells, the secretion of proinflammatory factors and the activation of macrophages, as well as ECM remodeling. If one compares the markers, CAFs, like some subtypes of FAPα-activated cells, express αSMA, vimentin, desmin, and FSP-1. An important role in maintaining the phenotype is assigned to the transmission of microRNAs in the composition of extracellular vesicles. Thus, microRNA-21 destroys the proapoptotic BAX and PTEN mRNA, reducing the sensitivity of fibroblasts to profibrotic stimuli [44]. Numerous studies indicate the important role of extracellular vesicles secreted by tumor cells in maintaining the CAF phenotype. Notably, increased matrix stiffness also appears to play a role in the survival of CAFs, as they have the same high capacity for ECM synthesis and remodeling as activated stromal cells in fibrosis [135].

To summarize, the activation and functions of fibroblasts during tumorigenesis and fibrogenesis have many common features, which suggests similar developmental mechanisms. Since FAPα is considered one of the key markers of CAFs from a number of tumors, as well as a marker of activated fibroblasts in fibrogenesis, by analogy, it is proposed to introduce the term fibrosis-associated fibroblasts (FAFs).

Therefore, some experimental results regarding CAFs could be approximated to FAFs. Specifically, information about the molecular partners of FAPα within the cell membrane of CAFs was partially verified for other activated fibroblasts. Thus, in recent years, there has been a growing recognition that the key role of FAPα in the regulation of cell signaling and differentiation may be determined by forming complexes with other proteins on the membrane, including organizing with them in lipid rafts at the border of invadopodia [136]. Chen and his colleagues were the first to identify invadopodia, membrane protrusions of invasive cells that contact and destroy ECM [58], and also showed that the presence of FAPα in the invadopodia determined the invasive phenotype of fibroblasts. It has been observed that blocking the protease activity of FAPα preserves the ability of FAPα^+^ cells to actively invade compared to cells not expressing FAPα [137]. Since previous studies have shown that FAPα in activated cells is localized to invadopodia, various integrins can be considered as putative partners for FAPα. Several studies indicate that FAPα can form complexes with β1 integrin, as evidenced by the coprecipitation of FAPα with α3β1 integrin [138]. For example, in a mouse model of lung cancer, tumors from FAPα-deficient animals showed increased forms of phospho-FAK (focal adhesion kinase) and phospho-ERK (extracellular signal-regulated kinases) compared to tumors from wild-type mice [139]. An increase in p21 expression was also observed in FAPα-deficient mice. The authors concluded that the deletion of FAPα increases p21 by enhancing FAK and ERK signaling [139], which are well-known downstream effectors of integrin signaling, and the enhanced migratory phenotype is mediated by integrin β1, as the addition of an integrin inhibitor reverses phenotypic changes [140]. Also, FRET data suggest that FAPα is in close proximity to the urokinase plasminogen activator receptor, uPAR [141]. uPAR promotes pericellular proteolysis by binding its specific ligand, the serine protease urokinase (uPA), which locally converts the ubiquitous zymogen plasminogen into active plasmin, a broad-spectrum protease that degrades ECM proteins either directly or through activating other proteases. Because uPAR lacks a transmembrane and cytosolic domain, signal transduction requires the interaction of uPAR with additional molecules on the cell surface. These may include receptor tyrosine kinases (such as EGFR, PDGFR) and integrins. Studies show that the binding of uPA to uPAR initiates the activation of intracellular signaling molecules such as FAK, mitogen-activated protein kinase (MAPK), and the Jak/Stat pathway, promoting actin cytoskeleton remodeling and cell migration. Based on these data, it can be assumed that the formation of FAPα–uPAR–integrin complexes will increase the efficiency of targeted pericellular proteolysis.

It is known that FAPα is able to form heterodimeric complexes with DPPIV and that these complexes promote cell migration [94]. There is evidence that mutant FAPα lacking enzymatic activity promotes cell migration [137]. It can be assumed that the role of FAPα here is to form heterodimers with DPPIV and to regulate/localize DPPIV activity. Other putative partners of FAPα include other membrane-bound proteases (MMP-2, MMP-14, uPA), as well as α3β1 integrins [61].

Taken together, the accumulating results of the studies exploring CAF properties provide useful implications for FAPα-positive cells involved in other pathologies. However, numerous data obtained for CAFs need to be tested for confirmation under fibrogenesis conditions utilizing FAPα-positive activated stromal cells.

## 7. Conclusions

By analyzing the available data, one can conclude that FAPα is not only a marker of activated stromal cells but is also responsible for the implementation of many functions associated with the development and progression of fibrosis. This information highlights the relevance of FAPα as a target for the therapy of fibrotic diseases. Indeed, today, a number of approaches have already been proposed in both fibrosis and theranostics based on the use of CAR-T [99,142], liposomes [143], or FAP inhibitors, including labeled ones for tracking the progression of treatment [144,145,146]. However, modern methods are aimed primarily at eliminating cells from the entire body and often lead to adverse consequences [74,77,147]. Using transgenic mice, it was shown that the systemic removal from the organism of cells expressing the FAPα gene led to the development of muscular dystrophy and cachexia. Also, in mice, after the elimination of FAPα-positive cells, erythropoiesis and lymphopoiesis are suppressed [74]. Similar data were obtained using CAR-T cells targeting FAPα-expressing cells [147]. In addition, the global deletion of FAPα in apolipoprotein E (ApoE) knockout mice has been shown to accelerate the progression of atherosclerosis [77].

**Figure 3:**
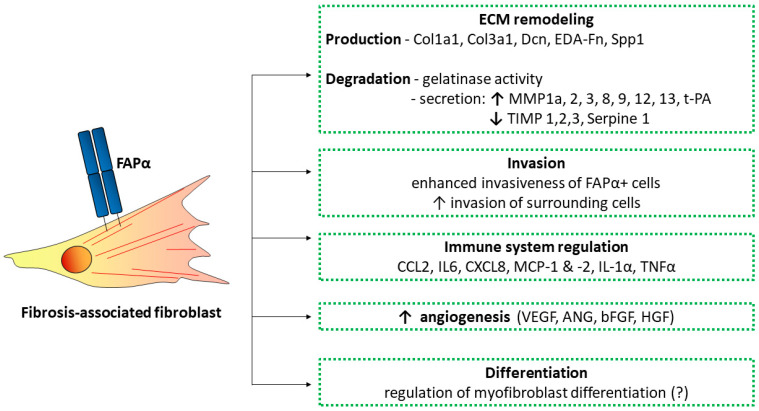
FAPα-positive fibroblasts may perform various functions during fibrosis progression and/or reversal. Partially, the mechanisms of these functions have already been revealed, while others—for example, the effect on myofibroblast differentiation—have yet to be explored. ↑—Function increase; ↓—decrease in function; ?—intended function.

Apparently, the cause of these adverse effects is the high expression of FAPα in local vital cell populations in various organs—for example, in bone marrow mesenchymal stromal cells (MSCs) [74]. Another reason for this phenomenon may be the different functions in subpopulations. FAPα^+^ cells are associated with fibrosis at different time points during the disease. As follows from the text above, in various models, the modulation of FAPα can lead to directly opposite effects (Figure 3), but the reasons for these phenomena are often unclear.

A number of studies note that FAPα-positive cells contribute to the progression of fibrosis and have a more proteolytically invasive phenotype at the stage of fibrosis progression and a secretory one when fibrotic tissue is already formed. At the same time, data indicate that these cells can also participate in the resolution of fibrosis in those models where it is possible—for example, in induced pulmonary fibrosis [98,99]. However, the exact mechanism of these phenomena still remains unclear. Nevertheless, there are already the first studies indicating the opposite roles of different subpopulations of FAPα-positive cells. It has been shown that the most important partner of FAPα, at least in inflammatory diseases, is CD90 (Thy-1). Interestingly, the role of Thy-1-positive or -negative cells in fibrosis has been studied for a long time. Thus, it has been shown that the loss of CD90 expression leads to the more severe development of pulmonary fibrosis in mice [148,149]. A number of articles indicate various mechanisms of Thy-1 involvement in fibrosis, including the regulation of autocrine TGF-β secretion in response to profibrogenic stimuli [150], changes in PDGFRα receptor expression [151], cells motility and mechanosensing [152,153], and extracellular vesicles uptake [154]. The coexpression of CD90 and FAPα was also presented in Tilmann’s work in the case of human myocardial infarction [78]. Thus, PDPN^+^FAPα^+^THY1^+^ cells play the role of an immune effector in the development of rheumatoid arthritis, capable of maintaining inflammation through the secretion of a different repertoire of chemokines and cytokines, as PDPN^+^FAPα^+^THY1^−^ cells are capable of regulating osteoclast behavior [76,155]. It is of note that the role of FAPα-expressing cells other than fibroblasts in the development of fibrosis and the pathogenesis of fibrotic diseases is elusive. Further lineage-tracing studies along with a deeper immunohistochemical and single-cell transcriptomic analysis of the subpopulation composition of fibrotic tissues in various organs are necessary to advance this field of research.

Therefore, in the authors’ opinion, the first priority is to reveal the exact mechanisms of the functioning of these cells, including those associated with FAPα partners. Understanding these mechanisms will allow for the local regulation of the necessary activity of FAPα-expressing cells, rather than eliminating the entire population. Thus, our studies have shown that the intratracheal administration of extracellular vesicles secreted by MSCs to mice in a model of bleomycin-induced lung fibrosis can reduce the number of FAPα-expressing cells in the lung tissue, which correlates with a decrease in the severity of fibrosis. In many ways, this effect may be mediated by the transfer of microRNAs—in particular, microRNAs-29c and 129—in extracellular vesicles, which affect the expression of many proteins associated with FAPα—for example, integrin α5 and integrin β1 [156,157]. However, the exact mechanisms of this phenomenon have not been shown. Another promising microRNA may be miR-30a, which directly suppresses the expression of FAPα, type 1 collagen, and αSMA induced by TGF-β [83].

Taken together, activated stromal cells expressing FAPα may definitely be the critical regulators of fibrosis development and progression. It can be suggested that FAPα may be the primary molecule responsible for accelerating the transition of stromal progenitor cells from an activated state to myofibroblasts promoting the pathogenesis of fibrosis. These properties make both FAPα- and FAPα-positive cells promising targets for developing novel antifibrotic approaches.
